# Long-Term Outcomes of Targeted Therapy after First-Line Immunotherapy in BRAF-Mutated Advanced Cutaneous Melanoma Patients—Real-World Evidence

**DOI:** 10.3390/jcm11082239

**Published:** 2022-04-17

**Authors:** Paweł Rogala, Anna M. Czarnecka, Bożena Cybulska-Stopa, Krzysztof Ostaszewski, Karolina Piejko, Marcin Ziętek, Robert Dziura, Ewa Rutkowska, Łukasz Galus, Natasza Kempa-Kamińska, Jacek Calik, Agata Sałek-Zań, Tomasz Zemełka, Wiesław Bal, Agnieszka Kamycka, Tomasz Świtaj, Grażyna Kamińska-Winciorek, Rafał Suwiński, Jacek Mackiewicz, Piotr Rutkowski

**Affiliations:** 1 Department of Soft Tissue/Bone Sarcoma and Melansoma, Maria Sklodowska-Curie National Research Institute of Oncology, 02-781 Warsaw, Poland; pan.rogal@gmail.com (P.R.); krzysztof.ostaszewski@pib-nio.pl (K.O.); tomasz.switaj@pib-nio.pl (T.Ś.); piotr.rutkowski@pib-nio.pl (P.R.); 2 Department of Experimental Pharmacology, Mossakowski Medical Research Centre, Polish Academy of Sciences, 02-106 Warsaw, Poland; 3 Department of Clinical Oncology, Maria Sklodowska-Curie National Research Institute of Oncology, Cracow Branch, 31-115 Kraków, Poland; bcybulskastopa@vp.pl (B.C.-S.); karolina.wojdyla@hotmail.com (K.P.); agatasalek@gmail.com (A.S.-Z.); tzemelka@mp.pl (T.Z.); 4 Department of Surgical Oncology, Wroclaw Comprehensive Cancer Center, 53-413 Wroclaw, Poland; zietekm@op.pl; 5 Department of Oncology, Wroclaw Medical University, 50-376 Wroclaw, Poland; 6 Department of Clinical Oncology, Holy Cross Cancer Center, 25-734 Kielce, Poland; onko.rdz@gmail.com (R.D.); ewarutkowskasco@gmail.com (E.R.); 7 Department of Medical and Experimental Oncology, University of Medical Sciences, 61-701 Poznan, Poland; galus.lukasz@spsk2.pl (Ł.G.); jmackiewicz@ump.edu.pl (J.M.); 8 Department of Clinical Oncology, Wroclaw Comprehensive Cancer Center, 53-413 Wroclaw, Poland; kempa.natasza@dco.com.pl (N.K.-K.); calik.j@dco.com.pl (J.C.); 9 Department of Chemotherapy, Maria Sklodowska-Curie National Research Institute of Oncology, Gliwice Branch, 44-102 Gliwice, Poland; wieslaw.bal@io.gliwice.pl; 10 Subcarpathian Oncology Center, 35-061 Rzeszów, Poland; agnelika.ka@gmail.com; 11 The Skin Cancer and Melanoma Team, Department of Bone Marrow Transplantation and Hematology-Oncology, Maria Sklodowska-Curie National Research Institute of Oncology, Gliwice Branch, 44-102 Gliwice, Poland; grazyna.kaminska-winciorek@io.gliwice.pl; 12 II Clinic of Radiotherapy and Chemotherapy, Maria Sklodowska-Curie National Research Institute of Oncology, Gliwice Branch, 44-102 Gliwice, Poland; rafal.suwinski@io.gliwice.pl; 13 Department of Diagnostics and Cancer Immunology, Greater Poland Cancer Centre, 61-866 Poznan, Poland

**Keywords:** melanoma, *BRAF*, dabrafenib, vemurafenib, encorafenib

## Abstract

Background: Currently, limited data on targeted therapy and immunotherapy sequencing in patients with *BRAF*-mutant melanoma is available. Targeted therapy and immunotherapy are expected to be comparable in terms of overall survival (OS) when used as second-line therapies; therefore, understanding the characteristics of patients who completed sequential treatment is needed. Methods: The primary objective of this study was to analyze the efficacy of BRAFi/MEKi activity as second-line therapy in patients with advanced melanoma. We also aimed to describe the clinical characteristics of patients with advanced melanoma who were treated sequentially with immunotherapy and targeted therapy. We enrolled 97 patients treated between 1st December 2015 and 31st December 2020 with first-line immunotherapy with programmed cell death 1 (PD-1) checkpoint inhibitors; and for the second-line treatment with at least one cycle of BRAFi/MEKi therapy with follow-up through 31 January 2022. Results: Median OS since first-line treatment initiation was 19.9 months and 12.8 months since initiation of BRAFi/MEKi treatment. All BRAFi/MRKi combinations were similarly effective. Median progression free survival (PFS) was 7.5 months since initiation of any BRAFi/MEKi treatment. Conclusions: BRAFi/MEKi therapy is effective in the second-line in advanced and metastatic melanoma patients. For the first time, the efficacy of all BRAFi/MEKi combinations as second-line therapy is shown.

## 1. Introduction

Currently, limited data is available on targeted therapy and immunotherapy sequencing in patients with B-Raf Proto-Oncogene (*BRAF*)-mutant melanoma. Some studies suggest lower activity of immunotherapy after BRAF/MEK inhibitors (BRAFi/MEKi), but there are limited data on BRAFi/MEKi after immunotherapy failure [1]. It was suggested that first-line treatment with targeted therapy might select aggressive melanoma clones and therefore limit the benefit of second-line immunotherapy [2]. Objective response rates (ORR) were lower for ipilimumab used after BRAFi failure in comparison to the reverse sequence [3]. Therefore an optimal treatment sequencing strategy for BRAF-mutated patients is still a matter of debate while final data from clinical trials like DREAMseq (Doublet, Randomized Evaluation in Advanced Melanoma Sequencing; ECOG-ACRIN EA6134; NCT02224781) and SECOMBIT (Sequential Combo Immuno and Target Therapy; NCT02631447) are awaited [4]. In general practice, clinicians’ decisions are based mostly on the clinical characteristics of particular patients and validated molecular biomarkers are not defined yet. The cost-effectiveness simulation of treatment in *BRAF-V600* mutant metastatic melanoma suggests starting treatment with anti-programmed cell death protein 1 (PD-1) plus anti- cytotoxic T-lymphocyte-associated protein 4 (CTLA-4) is more cost-effective than starting with targeted therapy (or anti-PD-1 monotherapy) [5].

Most patients with *BRAF*—mutated melanoma are treated with BRAFi/MEKi at some point in their therapy. BRAFi/MEKi are usually offered as first-line treatment for patients with high disease volume, elevated lactate dehydrogenase (LDH) activity, and/or with rapidly progressing disease and in patients who require rapid onset of therapy, including individuals with brain metastases [6]. Immunotherapy with anti-PD-1 antibodies (nivolumab, pembrolizumab) as well as a combination (nivolumab with ipilimumab) were shown to be effective regardless of *BRAF* mutation status [7,8]. At the same time, in a recent meta-analysis, the presence of a *BRAF* mutation was shown to be statistically significantly associated with reduced overall survival (OS) in metastatic melanoma patients [9]. Patients with advanced melanoma had a threefold increased risk of death over a 7.6-year follow-up period due to a more rapidly progressing disease compared with melanoma without *BRAF* mutations. Moreover, patients with BRAF mutated stage III melanoma had a 77% higher three-year recurrence rate [10,11]. Current studies need to include the treatment efficacy of new therapies in patients with malignant melanoma. The development of new treatment combinations urges additional clinical trials and their analysis in order to compare the efficacy of all available therapies. Possibly also combinations of BRAFi therapy and immune checkpoint inhibitors in patients with *BRAF*-mutated metastatic melanoma may change advanced melanoma treatment in the future [12].

Real-world data analysis may provide information on the efficacy of currently available combination therapies in a full spectrum of patients, including those who would not qualify for clinical trials. Moreover, only a limited number of reports are available to describe long-term treatment efficacy for patients with *BRAF* mutated melanoma, as—in general—trials offering treatment beyond the frontline setting are rarely designed. Currently, targeted therapy and immunotherapy are expected to be comparable in terms of OS when used as second-line therapies; therefore, understanding the characteristics of patients who completed sequential treatment is needed to identify those who are more likely to get long-term benefits from currently available melanoma therapies. Until now, no large volume of real-world data is available on sequencing treatments in patients with *BRAF*-mutated melanoma, including those with poor prognostic factors. Such data is necessary to support sequential therapy selection in everyday patient care. The primary objective of this study was to analyze the efficacy of BRAFi/MEKi activity as second-line therapy in patients with advanced melanoma. We also aimed to describe the clinical characteristics of patients with advanced melanoma who are treated sequentially with immunotherapy and targeted therapy.

## 2. Materials and Methods

### 2.1. Patients Analyze

For this observational study, we analyzed electronic health record data of adult patients who started first-line therapy for advanced/metastatic *BRAF*-mutated melanoma from 1 December 2015 to 31 December 2020, with follow-up through 31 January 2022. We have included all consecutive sequentially treated patients from major oncology centers in Poland who have been treated with first-line immunotherapy with an anti-PD-1 checkpoint inhibitor (nivolumab or pembrolizumab) and for the second-line treatment with at least one cycle of BRAFi/MEKi therapy. Patients were recruited as described before [13]. Patients with brain metastases were asymptomatic and did not require steroids (prednisone > 10 mg treatment when immunotherapy was started. All eligible patients had the diagnosis confirmed by pathologists experienced in skin cancer pathology and a confirmed *BRAF* mutation. Patients treated with neoadjuvant [14] and adjuvant therapies, treated after RECIST progression [15], as well as treated within clinical trials were excluded from the study.

### 2.2. Data Analysis

Patients’ characteristics were analyzed with descriptive statistics. Progression-free survival (PFS) and overall survival (OS) were calculated with the Kaplan–Meier method. Log-rank test was used to assess differences between survival curves. The Cox proportional hazard model was used for multivariable analysis. All variables with a *p*-value < 0.1 in univariate analysis were included in the multivariable model. 95% confidence intervals (CI) were reported. The differences were considered statistically significant if the *p*-values were <0.05 [16]. Patients without signs of disease progression (PD) were censored at the last follow-up visit. The OS was calculated from the date of treatment start to death or last follow-up. Analysis was performed with Statistica version 13.3.

## 3. Results

### 3.1. Patients Treated

The enrolled patients included 42 females and 55 males (Table 1) with a median age of 62 years (28 to 81 y.o.). The majority (96%) of patients started treatment with stage IV disease and among all patients, almost 20% presented with asymptomatic brain metastases at treatment initiation. More than 47% of patients had elevated LDH at the first-line treatment start. The median time from initiation of first-line therapy to initiation of second-line was 4.2 months (1.1–17.6 months), and the median time from initiation of second-line to time of death (or last visit) was 9.8 months (6.4–19.9). The median follow-up time from initiation of first-line therapy was over 18.2 months and after second-line therapy was 9.8 months.

### 3.2. Sequential Treatment

**Figure 1:**
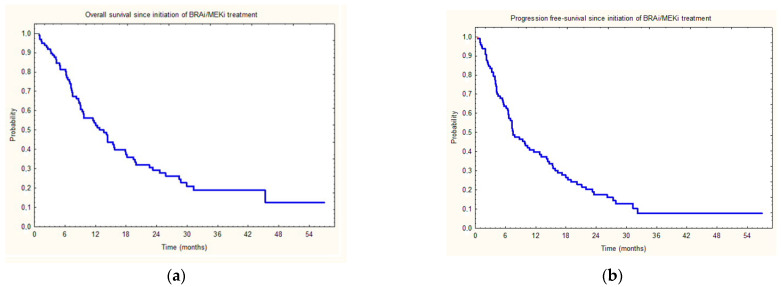
Overall survival (**a**) and progression-free survival (**b**) of melanoma patients since initiation of second-line BRAFi/MEKi treatment.

In the whole group, the first-line treatment used was nivolumab in 49 cases (51% of all treated patients) and pembrolizumab in 48 cases. Among patients treated with BRAFi/MEKi in the second-line, 17 patients continued BRAFi/MEKi treatment at the time of analysis. At data cut-off, 21 patients were referred to third-line treatment, 5 for BSC due to PD, and 69 patients died. Median OS since first-line treatment initiation was 19.9 months, and 12.8 months since initiation of BRAFi/MEKi treatment (Figure 1a). Median PFS on immunotherapy (first-line treatment) was 3.0 months and 7.5 months since initiation of BRAFi/MEKi treatment (Figure 1b).

**Figure 2:**
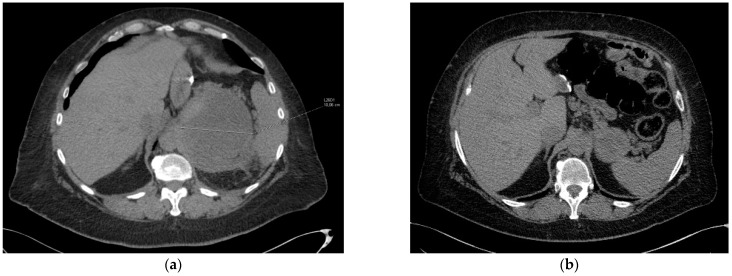
Partial response after BRAFi/MEKi treatment used in second-line. (**a**) CT scan from 28 December 2020, (**b**) CT scan from 1 April 2021.

**Figure 3:**
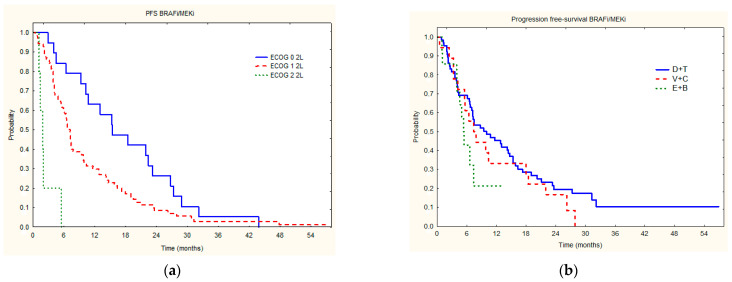
Median PFS on second-line BRAFi/MEKi treatment in relation to ECOG status (**a**) and drug used (**b**) (D + T—Dabrafenib + Trametinib; V + C—Vemurafenib + Cobimetinib; E + B—Encorafenib + Binimetinib).

There were no statistically significant differences in ORR between the treatment groups with different BRAFi/MEKi (*p* = 0.94) combinations (Table 2), median PSF (*p* = 0.40400) on BRAFi/MEKi, as well as median OS (*p* = 0.3879) on BRAFi/MEKi (Table 2, Figure 2 and Figure 3). PSF and OS on dabrafenib + trametinib were 9.4 and 14.3 months, on vemurafenib + cobimetinib—7.4 and 8.7 months, and for encorafenib + binimetinib—5.4 and 9.1 months, respectively. Groups treated with different BRAFi/MEKi combinations did not differ in subgroups based on the LDH level (*p* = 0.3068), gender (*p* = 0.70724), age (*p* = 0.9798) or presence of brain metastases (*p* = 0.37648). LDH level (*p* = 0.0463) correlated with median PSF in the whole group (Table 3). The presence of brain metastases (0.1164) did not correlate with median PSF in the whole group.

**Figure 4:**
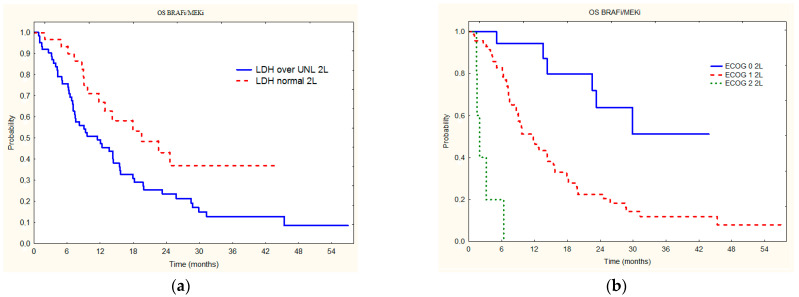
Overall survival on second-line BRAFi/MEKi treatment in relation to LDH levels (**a**) and ECOG status (**b**).

The factor that correlated with OS achieved after initiation of second-line treatment, both in univariate as well as multivariate analyses, was overall status according to the ECOG scale (*p* = 0.0001) (Figure 4, Table 4), while LDH was significant only in univariate analysis.

## 4. Discussion

Median progression-free survival of 7.5 months and overall survival after BRAFi/MEKi initiation of 12.8 months found in this study were comparable with data reported by other real-world studies, recent ongoing clinical trials (i.e., SECOMBIT), and cross subgroup analyses (Figure 1 and Figure 3). This real-world analysis utilizing the nationwide data from multiple reference oncology centers confirms that treatment with second-line BRAFi/MEKi therapy prolongs the survival of advanced/metastatic *BRAF*-mutated melanoma patients. In our study, the survival of patients who received sequential therapy was investigated. Our data is in agreement with initial observations from the phase II SECOMBIT trial. This trial provided the first prospective evidence of the optimal sequence of immune checkpoint inhibitor therapy and targeted treatment combination choice in patients with *BRAF*-mutated advanced melanoma. These authors have shown that treatment with ipilimumab plus nivolumab, then encorafenib plus binimetinib, or a ‘sandwich’ strategy improves survival rates (ESMO Congress 2021; LBA40). In fact, two- and three-year survival rates showed a positive trend in the ipilimumab plus nivolumab -> encorafenib plus binimetinib (arm B: 73% and 62%, respectively) sequence and with the sandwich strategy (arm C: 69% and 60%, respectively) compared with encorafenib plus binimetinib -> ipilimumab plus nivolumab (arm A: 65% and 54%, respectively). Similar effects were reported for two- and three-year PFS rates (arm B: 65% and 53%; arm C: 57% and 54%; arm A: 46% and 41%, respectively). Data on survival in this trial are still being collected. Our analysis has confirmed that encorafenib and binimetinib are effective treatment options in immunotherapy-pretreated patients (Figure 3) with PFS, similar to other BRAFi/MEKi combinations.

Current data from the phase III DREAMseq trial (NCT02224781), also known as the ECOG-ACRIN EA6134 trial, also confirmed BRAFi/MEKi efficacy as a second-line treatment. This trial evaluated dabrafenib/trametinib followed by nivolumab plus ipilimumab or vice versa sequential treatment. In these patients, after disease progression, response rates to second-line treatment were 48% with dabrafenib/trametinib and 30% with nivolumab/ipilimumab. In this trial, RR were similar for dabrafenib/trametinib used both in first or second-line, but the nivolumab plus ipilimumab combination appeared to be less effective after PD on dabrafenib/trametinib than in first-line therapy [17]. Our results indicate a similar trend of dabrafenib/trametinib efficacy as second-line therapy (Figure 3) in routine clinical practice.

A second-line analysis similar to ours was conducted on 79 patients with a median age of 60 years, 68% with stage M1c melanoma, 25% of whom had brain metastases, 57% had elevated LDH, and 24% were ECOG 2/3. In this analysis, 55 (70%) patients received combination BRAFi/MEKi, 22 (28%) BRAFi alone, and 2 (3%) MEKi alone. Among those 10/79 (13%) patients stopped taking BRAFiMEKi due to toxicity, 2—prior to first response, while median PFS was 4.4 months (3.5–6.2) and median treatment duration was 21 weeks. 59% of patients had a RECIST response (5% CR), 11% had SD, and 29% had PD as the best response [1]. The efficacy of BRAFi/MEKi as second-line therapy is also supported by reports showing that long-term metastatic melanoma treatment efficacy is affected by BRAFi exposition before treatment with pembrolizumab [2]. Similarly, in the Flatiron Health oncology-focused electronic medical record (EMR) database from the USA, at a mean follow-up of 15–16 months, second-line therapy was administered to 33 and 41% of patients treated in first-line with nivolumab plus ipilimumab or BRAFi/MEKi, respectively. Unfortunately, differences between the groups were observed in the prevalence of comorbidities (66 vs. 53%, respectively; *p* = 0.02), ECOG performance status (50 vs. 36%; *p* = 0.04), and LDH level (48 vs. 33%; *p* = 0.04), so it is not fully conclusive [18]. BRAFi/MEKi as second-line therapy provides higher ORR, as in a large retrospective analysis, metastatic melanoma patients resistant to PD-1 who were treated with PD-1 + CTLA4 achieved an ORR of 31%, while among those treated with CTLA4 monotherapy only 12% [19]. The study of pembrolizumab with ipilimumab as second-line therapy for advanced melanoma provided compelling evidence that the combination of PD-1 and CTLA4 is a safe and effective treatment approach in the PD-1–refractory patient population [20].

Real-word evidence following the randomized trials should better define the effectiveness of treatments in routine clinical practice, including subgroups of patients who are usually excluded or under-represented in the trials. Such analysis may provide clinically significant insights into second-line therapy in everyday real-world practice [21]. Moreover, in our study, we have shown for the first time that in routine clinical practice, all BRAF/MEKi combinations are effective as second-line therapy, as well as comparable in terms of PFS achieved by the patients (Figure 4b). Such data, as presented in our manuscript, are important and complement the results obtained from randomized controlled trials [21]. For cobimetinib plus vemurafenib used in previously untreated BRAFV600 mutation-positive advanced melanoma in the randomized phase III coBRIM study, median PFS was 12.6 months, while median OS was 22.5 months when analyzed with at least five years since the last patient randomization [22]. Dabrafenib and trametinib combination resulted in a median PFS of 11.1 months and a median OS of 25.9 months, and an overall survival rate of 34% at five years in patients treated in the COMBI-d (NCT01072175) and COMBI-v (NCT01597908) trials [23]. For the COLUMBUS trial (NCT01909453), the median PFS for patients treated with encorafenib 450 mg QD + binimetinib 45 mg BID was 14.9 months, while median OS and five-year OS rates were 33.6 months and 34.7%, respectively [24,25]. As described, the majority of registration trials enrolled treatment naïve patients, and little is known about the efficacy of BRAFi/MEKi as later lines of therapies and in patients that do not meet the trial inclusion criteria. PFS and OS resulting from later lines of treatment need to be defined by such studies from routine clinical practice. Our study fulfills, therefore the need for an analysis of pre-treated patients. Although network meta-analysis suggested that cobimetinib plus vemurafenib, dabrafenib plus trametinib, and encorafenib plus binimetinib are of similar efficacy as first-line therapy in advanced melanoma, these agents have not been compared in the second-line before [26].

Prognostic factors that we have found significant for second-line treatment efficacy are in accordance with these defined for post-progression OS after progression on treatment with cobimetinib plus vemurafenib, vemurafenib monotherapy, or dacarbazine in the BRIM-2 (NCT00949702), BRIM-3 (NCT01006980), BRIM-7 (NCT01271803), and coBRIM (NCT01689519) studies. It was shown that baseline lactate dehydrogenase, baseline disease stage, as well as ECOG performance status at progression, and second-line therapy usage are significant prognostic factors in such a clinical situation. Median post-progression OS was reported longest in patients with normal baseline LDH, M1c disease at baseline (no brain metastases), and second-line immunotherapy or targeted therapy; and on the contrary shortest in those with elevated baseline LDH > 2× upper limit of normal [27], which is also the case in our study. Also, in a dabrafenib plus trametinib compassionate-use setting, in patients with known brain metastases, ORR was 61.3%, median PFS was 6.2 months, and median OS was 15.5 months which is similar to our results [28]. In the whole DESCRIBE III program in patients with unresectable or metastatic melanoma lower LDH levels and <3 metastatic sites at baseline were associated with a longer duration of benefit from dabrafenib and trametinib therapy, which confirmed that the findings from COMBI-d and COMBI-v trials are also relevant to patients treated in a real-world setting [29], and this is also the case in our patients. As confirmed by us and other studies described, we believe that in routine clinical practice, patients’ baseline characteristics are of primary concern when selecting treatment sequences. More robust international analysis, combining our data with data from other countries could confirm the significance of prognostic factors shown in this study.

## 5. Conclusions

BRAFi/MEKi therapy is an effective second-line treatment in advanced and metastatic melanoma patients. Patients with ECOG 0 and 1 achieved significant benefits in terms of OS and PFS with this sequential treatment. BRAFi/MEKi therapy used in second-line enables induction of rapid response also in patients progressing after immunotherapy, including anti-PD1 monotherapy. The use of BRAFi/MEKi may prolong survival even after the failure of immunotherapy and the development of new metastases. The choice of drug combination selected for treatment should be based on patient preferences and the decision of the attending physician. It is important to await the results of randomized trials comparing an anti–PD-1 and anti-CTLA4 antibody combination before/after BRAFi/MEKi failure to make a definite statement of the superiority of any treatment sequence. Nonetheless, this study provides compelling evidence that the combination of BRAFi/MEKi is a safe and effective treatment approach in the anti-PD-1–refractory melanoma patient population in routine practice. Our study also provides evidence of sequential treatment efficacy in patients who did not start and/or do not qualify for the treatment with nivolumab and ipilimumab combination, including those with comorbidities and the elderly. In the future, additional molecular biomarker studies should be designed in order to support treatment choice decisions. A potential future area of investigation could also examine the effectiveness of second-line ipilimumab in *BRAF-mutated* melanoma patients after progression on first-line nivolumab or pembrolizumab and the comparative effectiveness between ipilimumab monotherapy and BRAFi/MEKi combination. If possible, patients progressing on first-line therapy should be referred for ongoing clinical trials and consulted by a multidisciplinary team.

## Figures and Tables

**Table 1:** Baseline patient characteristics.

Factor		No of Patients *n* = 97	% of Patients
Sex	F	42	43
	M	55	57
Age	≤65 years	64	66
	>65 years	33	44
Disease stage 1L	Locally advanced	4	4
	M1a	22	23
	M1b	16	16
	M1c	36	37
	M1d	19	20
LDH 1L	Normal	51	53
	Over ULN	46	47
ECOG 1L	0	47	48
	1	50	52
	2	0	0
Liver metastases 1L	No	77	79
	Yes	20	21
Brain metastases 1L	No	78	80
	Yes	19	20
First-line treatment	Nivolumab	49	51
	Pembrolizumab	48	49
	Nivolumab + ipilimumab	0	0
Second-line treatment	Dabrafenib + trametinib	65	67
	Vemurafenib + cobimetinib	18	19
	Encorafenib + binimetinib	14	14
LDH 2L	Normal	31	32
	Less than 2× over ULN	39	40
	More than 2× over ULN	23	24
	No data	4	4
ECOG 2L	0	19	20
	1	70	72
	2	5	5
	No data	3	3
Liver metastases 2L	No	63	65
	Yes	34	35
Brain metastases 2L	No	64	66
	Yes	33	34

No—number; F-female; M-male; ECOG—Eastern Cooperative Oncology Group performance status; 1L—first-line treatment; 2L—second-line treatment; LDH—lactate dehydrogenase; ULN—the upper limit of the norm.

**Table 2:** Best response on two lines of treatment.

Treatment Response		No of Patients	% of Patients
Best response immunotherapy *n* = 97	PD	57	59
	SD	24	25
	PR	13	13
	CR	2	2
	Not assessed	1	1
Best response BRAFi/MEKi *n* = 97	PD	20	21
	SD	20	21
	PR	51	52
	CR	5	5
	Not assessed	1	1
Best response Dabrafenib + Trametinib *n* = 65	PD	13	20
	SD	12	18
	PR	35	54
	CR	4	6
	Not assessed	1	2
Best response Vemurafenib + Cobimetinib *n* = 18	PD	4	22
	SD	4	22
	PR	10	56
	CR	0	0
	Not assessed	0	0
Best response Encorafenib + Binimetinib *n* = 14	PD	3	21
	SD	4	29
	PR	6	43
	CR	1	7
	Not assessed	0	0

PD—disease progression; SD—disease stabilization; PR—partial response; CR—complete response.

**Table 3:** Factors that influence median PFS on second-line BRAFi/MEKi treatment in advanced metastatic melanoma patients.

	Univariate Analysis			Multivariate Analysis		
Factor	HR	CI	*p*	HR	CI	*p*
Age (≤65 vs. >65 years)	0.8	0.5–1.3	0.4478	0.7	0.4–1.3	0.2787
Sex	0.8	0.5–1.3	0.4292	0.9	0.6–1.4	0.6528
LDH over ULN 2L	0.6	0.4–1.0	0.0496	0.8	0.4–1.3	0.3322
ECOG 2L 0 vs. 2	0.03	0.01–0.1	<0.0001	0.1	0.02–0.2	<0.0001
ECOG 2L 1 vs. 2	0.1	0.03–0.2	0.0183	0.1	0.04–0.3	0.0390
Brain metastases 2L	0.7	0.5–1.1	0.1443	0.7	0.5–1.2	0.2355
Liver metastases 2L	0.8	0.5–1.3	0.3279	1.0	0.6–1.7	0.9515

No—number; F-female; M-male; ECOG—Eastern Cooperative Oncology Group performance status; 1L—first-line treatment; 2L—second-line treatment; ULN—the upper limit of the norm; HR—hazard ratio; CI—confidence interval.

**Table 4:** Factors that influence OS in metastatic melanoma patients treated with immunotherapy—BRAFi/MEKi treatment sequence and on second BRAFi/MEKi.

Factor	Univariate Analysis			Multivariate Analysis		
	HR	CI	*p*	HR	CI	*p*
Age(≤65 vs. >65 years) OS 1L	0.9	0.5–1.4	0.5825	0.8	0.4–1.3	0.3270
Age(≤65 vs. >65 years) OS 2L	0.9	0.5–14	0.5477	0.8	0.5–1.5	0.5252
Sex OS 1L	0.8	0.5–1.3	0.3499	0.9	0.6–1.5	0.7109
Sex OS 2L	0.8	0.5–1.3	0.3468	0.9	0.5–1.5	0.6147
LDH over ULN 1L	0.8	0.5–1.3	0.3833	0.9	0.6–1.5	0.7943
LDH over ULN 2L	0.5	0.3–0.9	0.0220	0.7	0.4–1.2	0.1932
Brain metastases 1L	0.9	0.5–1.5	0.6465	0.8	0.4–1.5	0.5024
Liver metastases 1L	0.7	0.4–1.2	0.2295	0.7	0.4–1.4	0.3338
Brain metastases 2L	0.7	0.4–1.1	0.1164	0.8	0.5–1.3	0.2952
Liver metastases 2L	0.7	0.4–1.1	0.0941	0.9	0.5–1.7	0.7908
ECOG 2L 0 vs. 1 1L	0.6	0.4–0.9	0.0276	0.6	0.4–1.0	0.0620
ECOG 2L 0 vs. 2 2L	0.02	0.01–0.08	<0.0001	0.02	0.01–0.1	<0.0001
ECOG 2L 1 vs. 2 2L	0.1	0.03–0.25	0.1438	0.1	0.03–0.3	0.0889

No—number; OS—overall survival; ECOG—Eastern Cooperative Oncology Group performance status; 1L—first-line treatment; 2L—second-line treatment; ULN—the upper limit of the norm; HR—hazard ratio; CI—confidence interval.

## Data Availability

All data are available for research cooperation purposes from the PI of the study upon Data Transfer Agreement (DTA) approval (Piotr.Rutkowski@pib-nio.pl).
